# Infectious and non-infectious diseases burden among Haitian immigrants in Chile: a cross-sectional study

**DOI:** 10.1038/s41598-020-78970-3

**Published:** 2020-12-17

**Authors:** Francisco Fuster, Felipe Peirano, José Ignacio Vargas, Francisco Xavier Zamora, Marcelo López-Lastra, Ruth Núñez, Jacinta Soza, Katherine González, Denisse Estay, Beatrice Barchiesi, Antonieta Fuster, Ignacia López, Nicolás Utrera, Jorge Landeros, Javiera Chandía, Angela Paredes, Daniela Reyes, Rodrigo Arias, Luis Padilla, Hernán Suárez, Katia Farcas, Macarena Cannistra, Geraldine Muñoz, Ignacio Rodríguez, Ivana Ormazábal, Josefina Cortés, Bárbara Cornejo, Franco Manzur, Antonia Reyes, Vicente Leiva, María Victoria Raimann, Catalina Arrau, Valentina Cox, Alejandro Soza

**Affiliations:** 1 Hepatology Unit. Hospital Gustavo Fricke. Viña del Mar, Valparaíso, Chile; 2 Faculty of Medicine, Universidad de Valparaíso, Valparaíso, Chile; 3 Department of Gastroenterology, Faculty of Medicine, Pontificia Universidad Católica de Chile, Diagonal Paraguay 362, of. 423, 8330077 Santiago, Chile; 4 Department of Infectology. Hospital Barros Luco Trudeau, Universidad de Santiago de Chile, Santiago, Chile; 5 Laboratorio de Virología Molecular, Instituto Milenio de Inmunología e Inmunoterapia. Departamento de Enfermedades Infecciosas e Inmunología Pediátrica. Escuela de Medicina, Pontificia Universidad Católica de Chile, Santiago, Chile; 6 Department of Gastroenterology, Universidad de Chile, Santiago, Chile; 7 School of Medicine, Universidad Andrés Bello, Santiago, Chile; 8 School of Medicine. Pontificia, Universidad Católica de Chile, Santiago, Chile; 9 School of Medicine, Universidad del Desarrollo, Santiago, Chile

**Keywords:** Hepatitis, HIV infections, Population screening

## Abstract

Chile has become a popular destination for migrants from South America and the Caribbean (low- and middle-income countries migration). Close to 200.000 Haitian migrants have arrived in Chile. Infectious and non-infectious disease burden among the Haitian adult population living in Chile is unknown. This study aimed to acquire the basic health information (selected transmissible and non-transmissible conditions) of the Haitian adult population living in Chile. A cross-sectional survey was performed, inviting Haitian-born residents in Chile older than 18 years old. Common conditions and risk factors for disease were assessed, as well as selected transmissible conditions (HIV, HBV, and HCV). 498 participants (60.4% female) from 10 communities in two regions of Chile were surveyed. Most subjects had never smoked (91.5%), and 80% drank less than one alcohol unit per month. The mean BMI was 25.6, with 45% of participants having a normal BMI (20–25). Hypertension was present in 31.5% (33% in the 25–44 age group). Prevalence of HIV was 2.4% (95 CI 1.3–4.2%), hepatitis B (HBsAg positive) was 3.4% (95 CI 2.1–5.5%), and hepatitis C was 0% (95 CI 0.0–0.9%). Quality of life showed a significant prevalence of depression and anxiety markers, particularly in those arriving in Chile less than 1 year ago. Low prevalence of obesity, diabetes, smoking, and drinking and estimated cardiovascular risk were found. Nonetheless, hypertension at a younger age, disproportionately higher prevalence of HIV and HBV infection and frequent markers of anxiety and depression were also found. Public policies for detecting and treating hypertension, HIV, and HBV screening, offering HBV vaccination, and organizing mental health programs for Haitian immigrants, are urgently needed.

## Introduction

International migration is a universal phenomenon, representing a significant opportunity for economic, cultural, and social integration. Even though global news tends to highlight refugee crises and migration-related political issues in affluent countries, 42% of migrants live in developing countries. The so-called South-South migration (among low or middle-income countries) comprises 36% of the total migration, representing a greater rate than the South-North migration^1,2^.

Political and economic stability has positioned Chile as an attractive destination for South-South migration in the last decade. According to data aggregated by the International Organization for Migration, international migrants as a fraction of the local population is 3.5% for the World and 1.8% for Latin America. In Chile, international migrants increased from 1.7% of the total population in 2005 to 5% in 2019^3^. This growth is faster than anywhere else in Latin America, making Chile a key player in the recent migration movements in Latin America^4^. At the end of 2018, an estimated 1,251,225 foreign-born residents were living in Chile, a figure second only to Argentina in the region. Among these, 179,338 were born in Haiti, with most of them arriving after 2015^5^.

The Haitian diaspora is explained by years of political instabilities coupled with frequent natural disasters, such as the 7.0 magnitude earthquake in 2010, which left 300.000 people dead and 1.6 million homeless, along with a cholera outbreak^6^. The Mathew hurricane in 2016 had a toll of 3,000 deaths, infrastructure damage, and new cholera outbreaks^7^. Two million Haitians have migrated in the last decade, with three well-defined patterns of migration: flux "A" to France, Canada, and United States (the wealthier and more educated), flux "B" to Brazil and Chile, and flux "C" to the nearby Dominican Republic (the most precarious migrants)^8,9^.

Research regarding the health status of migrants in Chile possesses several barriers: Absence of studies specifically designed to address migrants populations, an inaccurate registration of the nationality or country of origin in the host country health system and clinical records, difficulty in approaching migrants due to language barriers and a natural fear of deportation and the absence of vigilance/monitoring of the living and health conditions of migrants by the host community^10^. Additional complexity is the implementation of the necessary extra safeguards required when dealing with a highly vulnerable population. This partially explains why, today, the information regarding migrant's health status in Chile is scarce. To date, few studies have been conducted to address immigration issues by either public agencies, non-governmental organizations (NGOs), or universities. The lack of information has created a critical knowledge gap that has only served to increase discrimination toward the migrant population. Therefore, it is paramount to understand the specific needs of the incoming migrant populations to promote the development of novel public policies directed towards their wellbeing, as a significant step for their adequate integration in the hosting society.

The Haitian community was selected for this study as their integration into the Chilean society has been highly hampered by language (Creole vs. Spanish), customs, community social structure, and ethnicity. This study aimed to acquire the basic health information (selected transmissible and non-transmissible conditions) of the Haitian adult population living in Chile. We hypothesized that Haitian-born subjects living in Chile exhibited a similar prevalence of health conditions as found in their country of origin. Therefore, we expected this migrant population's overall health status to sharply differ from the health status of the general Chilean population.

## Methods

### Study design

A cross-sectional survey was conducted among Haitian-born adult subjects living in Chile. The survey included a structured interview, anthropometric measurements, and a blood sample. The survey instrument included selected questions from the Chilean National Health Survey^11^. Before the definitive study protocol, the proposal was presented and approved by several Haitian community leaders in Chile and to NGOs working with migrants. The study protocol was approved by the Ethical and Scientific Committee of the Faculty of Medicine of the Pontificia Universidad Católica de Chile (CEC-MedUC, project ID 170614001) and was carried out in accordance with relevant guidelines and regulations, including the declaration adopted by the 18th World Medical Assembly (Helsinki, 1964) and subsequent amendments, and local regulations. All subjects were informed about the survey with the help of creole speaking cultural mediators, with an especially created video explaining the study in creole (https://youtu.be/fz69YErEBjc) and with the consent document itself.

### Setting

The survey took place between January 2018 and September 2019. The study was carried out in two Chilean regions (Región Metropolitana and Región de Valparaíso), the two most populous areas in Chile. Haitians from 10 different urban localities of these two regions were invited to participate by approaching local community leaders in churches and municipal centers. Surveys were carried out during weekdays and also during weekends to avoid selection bias for actively working subjects. A written survey (in creole) was responded by participants with the help of a cultural mediator. After their participation, subjects received breakfast and a small amount of money for transportation expenses (equivalent to US$3–4). Blood samples were processed by the laboratory the same day, except for Sundays, when samples were centrifuged and refrigerated to be analyzed the following day.

### Participants

Inclusion criteria included being born in Haiti, living in Chile (regardless of their migration status), being over 18 years old, being fasted for 8–12 h, and signing the informed consent. Pregnant women were allowed to participate in the survey. Prospective participants were asked to take part in the research program, with no implicit or explicit suggestion that they would receive medical treatments or interventions, which could bias selection for people with known health problems. Similarly, it was made clear that participation in this research program that, if diagnosed or suspected of any health-related condition, this information would not affect their migration status or job eligibility in Chile.

### Variables

Demographic data included age, gender, the nationality of both parents, origin (urban or rural), time in Chile, difficulty understanding Spanish, level of studies (primary, secondary or superior), health insurance in Chile (public, private or no insurance), job status during the last month, pregnancy status for females, recalling having ever received a vaccine. Regular status was defined as having a national ID number issued by the government.

The following measurements and variables were included: (1) Hypertension. A measurement with a calibrated automatic blood pressure monitor was performed after sitting for at least 5 min (before the blood sample was taken). Hypertension was defined as a systolic blood pressure > 140 mmHg and/or diastolic blood pressure > 90 mmHg. Patients with a self-reported diagnosis of chronic hypertension and patients receiving treatment for hypertension were also considered to have hypertension. (2) Lipids. Total, LDL, HDL cholesterol, and triglycerides were measured in plasma after fasting for 8–12 h. (3) Nutritional status. Weight, height, and waist circumference were measured according to the WHO recommendations^12^. Body mass index (BMI) was calculated, dividing the weight in kilograms by the square of the height in meters. An abdominal circumference > 88 cm in women and > 102 cm in men was considered increased. Waist circumference in pregnant women was excluded from the analysis. Self-reported nutritional status was also asked. (4) Diabetes mellitus. A fasting plasma glucose level was obtained. Abnormal glucose was defined as ≥ 100 mg/d but < 126 mg/dL and diabetes as ≥ 126 mg/dL and/or self-report of diabetes. (5) Smoking. (6) Alcohol consumption. (7) Quality of life. (8) Mood and depression. (9) Physical activity. (10) Renal function. Plasma creatinine was measured, and the glomerular filtration rate (GFR) was calculated with the CKD-EPI tool^13^. (11) HIV. The Abbott ARCHITECT HIV Ag/Ab Combo Assay, a fourth-generation assay, was used to diagnose HIV infection. All positive results were confirmed by a second sample. (12) Hepatitis C virus (HCV) infection. A microparticle immunoassay (chemiluminescent magnetic microparticle immunoassay; CMIA-Abbott ARCHITECT anti-HCV) in serum was used. Positive results were confirmed with an assay for RNA in plasma (Roche COBAS Real-Time PCR) in the laboratory of the ISP (*Instituto de Salud Pública).* (13) Hepatitis B virus (HBV) infection. Hepatitis B surface antigen (HBsAg) was determined in serum (CMIA-Abbott ARCHITECT HBsAg). Total anti-hepatitis B core (anti-HBc) antibodies were also determined in serum (CMIA-Abbott ARCHITECT anti-HBc). Samples testing positive for total anti-HBc were also tested for anti-HBc IgM in serum (CMIA-Abbott ARCHITECT anti-HBc IgM). (14) Cardiovascular risk was assessed according to the American College of Cardiology/American Heart Association atherosclerotic cardiovascular (ASCVD) risk estimator^14,15^.

### Report of individual results, linkage to primary care, and follow up

Individual results were handed to the participants, along with recommendations, 2 to 3 weeks after enrollment. Migrants in Chile are entitled to receive medical care (irrespective of their migratory status), so we teamed with different municipalities to enroll subjects in the public health care system (*Fondo Nacional de Salud*, Fonasa). To this end, a social worker from the Municipality invited participants who were not enrolled in Fonasa, obtaining fingerprints, generating a provisory health ID number, and linking the subjects to the respective Family Health Center (*Centro de Salud Familiar*, CESFAM). Transmissible diseases were notified to the Health Authority, as required by law, and chronic conditions such as the suspicion of hypertension or diabetes were notified to the subjects, as required by the Regime of Explicit Guarantees in Health Law^16^. All participants with confirmed or suspected diagnoses were followed up telephonically to ensure that their conditions were being treated.

### Sample size and statistical methods

Sampling was not probabilistic, rather based on response to an invitation (convenience sample). The sample size was determined based on an estimated prevalence of HIV infection in this population of 2.5%^17^ and 30% of hypertension. These conditions were selected given that Haiti is the country outside Africa with the highest HIV prevalence and that hypertension is more prevalent in the African descendant population, representing a common risk factor for cardiovascular disease. With this prevalence, 500 subjects would yield a 95% confidence interval (95% CI) between 1.3 and 4.1% for HIV. For hypertension, the 95% CI was 26 to 34%.

For the statistical analysis, frequency tables, proportions, and 95% confidence intervals (95% CI) were obtained for categorical variables using the modified Wald method^18^. Means, medians, standard deviation (SD), and interquartile ranges were calculated for continuous variables. The prevalence of selected conditions was stratified by age, sex, time since arrival in Chile, and educational level. Categorical variables were analyzed by contingency tables with the Fisher's exact test (two tails).

The denominators from which proportions or percentages were calculated are presented in the tables for a more transparent presentation of completeness of data (n/N). No imputation was used for missing data.

### Bias reduction

Efforts for reducing potential bias included the following measures: (1) Including subjects from 10 different communities in two different regions of the country. (2) Inviting subjects attending community centers and churches, as opposed to selecting patients attending health care centers. (3) Avoiding the expectative of getting treatment when participating in the study. (4) Minimizing monetary compensation (just reimbursement of transportation). (5) Ensuring the confidentiality of the health information for avoiding fear of deportation.

## Results

Participants from ten communities (20 to 94 subjects per site) in two regions of Chile participated in the survey, totaling 499 subjects. Approximately 5% of responders were not included in the study because they were not fasting. In addition, 2 individuals were excluded because they were younger than 18 years old. Among the enrolled participants, 29 did not have a blood sample taken (4 because of difficult vein access, and 25 because they refused the exam but agreed to have all other procedures).

The socio-demographic features of the enrolled participants are summarized in Table 1. There was a predominance of females (60.4%). In terms of age, most were between 25 and 44 years old. Noteworthy, participants' age distribution was similar to what has been described for the general population of Haitian immigrants in Chile. However, our data slightly over-represented women^5,8^.

**Table 1:** Socio-demographic characteristics of subjects.

Variable	
Female sex—n (%)	301/498 (60.4)
**Age**	
Mean ± SD	31.4 ± 7.6
18–24 years old—n/N (%)	79/498 (15.9)
25–44 years old—n/N (%)	365/498 (73.3)
45 years or older— n/N (%)	54/498 (10.8)
**First language—n/N (%)**	
Creole	484/498 (97.8)
French	8/498 (1.7)
Spanish	2/498 (0.4)
English	1/498 (0.2)
Difficulty speaking Spanish—n/N (%)	333/496 (67.1)
**Educational level—n/N (%)**	
Basic	108/489 (22.0)
Middle	204/489 (41.7)
Superior	177/489 (36.2)
Time in Chile (months)—median (IQR)	18 (8—24)
**Place of birth in Haiti—n/N (%)**	
Urban	165/489 (33.7)
Rural	324/489 (66.2)
**Last residence in Haiti—n/N (%)**	
Urban	246/492 (50.0)
Rural	246/492 (50.0)
Number of people living in the house—median (IQR)	3 (2—4)
**Marital status—n/N (%)**	
Single	59/85 (69.4)
Married	25/85 (28.0)
Widowed	0/85 (0.0)
Divorced	0/85 (0.0)
Cohabiting	1/85 (1.2)
Currently pregnant (among women)—n/N (%)	27/301 (9.0)
Type of health insurance*—n/N (%)	
FONASA (public system)	379/494 (76.7)
ISAPRE (private system)	2/494 (0.4)
No health insurance	113/494 (22.9)
**Having a job during the last month—n/N (%)**	
Yes, every day	163/496 (32.9)
Some days	52/496 (10.9)
No	223/496 (45.0)
Not looking for a job (housewife or other)	58/496 (10.9)

SD: Standard deviation, IQR: Interquartile range.

*FONASA: Fondo Nacional de Salud. ISAPRE: Institución de Salud Previsional.

Most had secondary or higher educational levels. They were predominantly born in rural areas, and their median stay in Chile was 18 months. Nine percent of the women were pregnant. Among subjects, 22.9% lacked any form of health insurance at the time of recruitment. Noteworthy, the public health system (FONASA) provides coverage to foreign individuals living in Chile, irrespective of their migration status. As a way of assisting participants, all subjects were enrolled in FONASA and referred to their respective health family center. Importantly, 55.9% of the participants were either unemployed or had worked only temporarily at the survey time. Only 59% (294 out of 498) of subjects had a regular migratory status.

Table 2 describes the prevalence of selected non-transmissible conditions and anthropometric measures. Most subjects (91.5%) had never smoked, and 80% did not drink or drank less than one alcohol unit per month. Sedentarism, defined as performing less than 3 moderate- and high-intensity physical activities per week, was 87.9%. Nonetheless, work-related physical activities were not captured by the survey. The mean BMI was 25.6. Nutritional status, evaluated by BMI, was normal in 45% of participants. Women tended to have a higher proportion of obesity and increased abdominal circumference than men. Remarkably, 9.1% of men were underweight (BMI < 20). The prevalence of hypertension was 31.5%, and specifically in the age group 25 to 44 years old, it was 33%. Only 16% were aware of having a diagnosis of hypertension. Suspicion of diabetes mellitus was very low, at 3%. Total cholesterol above 200 mg/dL was found in 13.9%. Only 0.2% of participants had an estimated glomerular filtration rate below 60 mL/min. Ten-year cardiovascular risk assessed by the ASCVD Risk Estimator was low (defined as < 5%) in 99.1% of participants. Given that the 10-year risk is more suited for subjects 40 and older, the lifetime cardiovascular risk was also calculated.

**Table 2:** Prevalence of selected conditions and anthropometry.

Condition	Total	Women	Men
**Smoking—n/N (%)**			
Yes, one or more per day	11/494 (2.2)	3/300 (1.0)	8/194 (4.1)
Yes, less than 1 per day	10/494 (2.0)	4/300 (1.3)	6/194 (3.1)
No, quitted	21/494 (4.3)	7/300 (2.3)	14/194 (7.2)
No, never smoked	452/494 (91.5)	286/300 (95.3)	166/194 (85.6)
Number of cigarettes per day in smokers—mean + SD	6.5 ± 5.2	7.5 ± 3.3	6.2 ± 2.0
Any alcohol drink in the last 12 months—n/N (%)	209/495 (42.2)	79/300 (26.3)	128/195 (51.2)
**Alcohol consumption—n/N (%)**			
Daily	8/491 (1.7)	5/299 (1.7)	3/192 (1.6)
5–6 drinks per week	10/491 (2.0)	3/299 (1.0)	8/192 (4.2)
1–4 drinks per week	18/491 (3.6)	3/299 (1.0)	14/192 (7.3)
1–3 drinks per month	62/491 (12.6)	19/299 (6.4)	43/192 (22.4)
< 1 drink per month	393/491 (80.0)	269/299 (90.0)	124/192 (64.6)
Number of drinks per day when drinking—mean	2.5	2.5	2.6
Sedentarism (physical activity < than 3 times per week)—n/N (%)	434/493 (88.0)	272/297 (91.6)	162/196 (82.3)
Height (m)—median (IQR)	1.66 (1.60–1.72)	1.62 (1.58–1.65)	1.73 (1.69–1.78)
**Nutritional status—n/N (%)**			
Low weigh (BMI < 20)	32/498 (6.4)	14/301 (4.7)	18/197 (9.1)
Normal (BMI 20–25)	224/498 (45.0)	104/301 (34.6)	120/197 (60.9)
Overweight (BMI 25–30)	159/498 (31.9)	107/301 (35.5)	52/197 (26.4)
Obesity (BMI 30–40)	77/498 (15.5)	70/301 (23.3)	7/197 (3.6)
Morbid obesity (BMI > 40)	6/498 (1.2)	6/301 (2.0)	0/197 (0.0)
Increased abdominal circumference—n/N (%)	124/469 (26.4)	119/273 (43.6)	5/196 (2.6)
**Blood pressure—n/N (%)**			
Hypertension	157/498 (31.5)	79/301 (26.2)	78/197 (39.6)
Systolic blood pressure ≥ 160 mmHg and/or diastolic blood pressure ≥ 110 mmHg	22/498 (4.4)	12/301 (4.0)	10/197 (5.1)
**Glucose and diabetes mellitus—n/N (%)**			
Abnormal glucose (100–126 mg/dL)	53/467 (11.3)	29/281 (10.3)	24/186 (12.9)
Diabetes mellitus	14/469 (3.0)	11/283 (3.9)	3/186 (1.6)
Glycemia ≥ 150 mg/dL	4/467 (0.9)	3/281 (1.2)	1/186 (0.5)
**Lipids—mean ± SD**			
Total cholesterol	162.3 ± 35.8	165.3 ± 35.0	157.8 ± 36.7
HDL cholesterol	50.7 ± 12.9	52.4 ± 13.3	48.6 ± 11.9
LDL cholesterol	95.7 ± 30.2	97.2 ± 28.6	93.6 ± 32.5
Triglycerides	79.0 ± 41.6	79.8 ± 43.8	77.8 ± 38.2
**Abnormal lipids—n/N (%)**			
Total cholesterol ≥ 200 mg/dL	65/469 (13.9)	45/282 (16.0)	20/187 (10.7)
HDL cholesterol < 40 mg/dL (m) or < 50 mg/dL (w)	178/469 (38.0)	132/282 (46.8)	46/187 (24.6)
LDL cholesterol ≥ 160 mg/dL	16/469 (3.4)	8/282 (2.8)	8/187 (4.2)
LDL cholesterol ≥ 190 mg/dL	4/469 (0.9)	2/282 (0.71)	2/187 (1.1)
Triglycerides ≥ 150 mg/dL	31/469 (6.6)	20/282 (7.1)	11/187 (5.9)
**Renal function**			
Creatinine—mean ± SD	0.79 ± 0.20	0.67 ± 0.13	0.95 ± 0.15
Estimated GFR < 60 mL/min—n/N (%)	1/469 (0.2)	1/282 (0.3)	0/187 (0.0)
**Cardiovascular risk**			
10-year risk (%)—mean ± SD	1.16 ± 1,26	1.01 ± 0.13	1.39 ± 1.98
10-year risk ≥ 5%—%	4/406 (0.99)	0/246 (0)	4/160 (2.5)
Lifetime risk (%)—mean ± SD	28.6 ± 14,43	25.0 ± 12.0	34.1 ± 16.0
**Self-report of conditions—n (%)**			
Hypertension	79/494 (16.0)	58/299 (19.4)	21/195 (10.8)
Diabetes mellitus	9/496 (1.8)	8/301 (2.7)	1/195 (0.5)
Hyperlipidemia	22/492 (4.5)	17/298 (5.7)	5/194 (2.6)
Unresolved health problem	168/494 (34.0)	107/492 (21.7)	61/195 (31.3)

SD: Standard deviation, IQR: Interquartile range.

**Figure 1:**
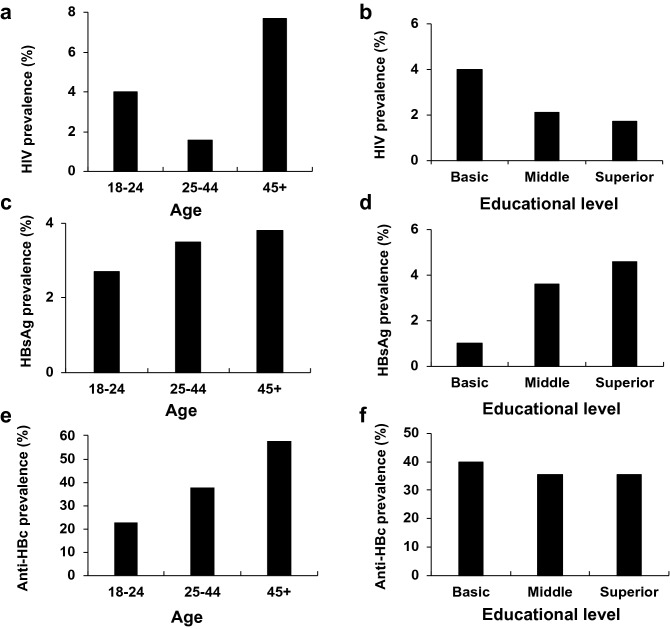
Prevalence of HIV infection, HBsAg and total anti-HBc stratified by age (**a**, **c** and **e**) and educational level (**b**, **d** and **f**).

The prevalence of human immunodeficiency virus (HIV), hepatitis B virus (HBV), and hepatitis C virus (HCV) among the studies population are detailed in Table 3. HIV prevalence (confirmed results) was 2.4% (95% CI 1.3–4.2), higher in women (3.2%), increased with age (Fig. 1a), and most infected individuals had only a basic level of education (4%) (Fig. 1b). HBV infection prevalence (HBsAg positive subjects) was 3.4% (95% CI 2.1–5.5), higher in men (4.3%), it increased with age (Fig. 1c), and mostly in subjects with higher educational levels (Fig. 1d). Exposure to the HBV, assessed by determining the total anti-HBc antibody, was 36.5%, and the prevalence for anti-HBc increased progressively with age (Fig. 1e). However, in contrast to the HBsAg (Fig. 1d), the anti-HBc antibody did not relate to the individual's educational levels (Fig. 1f). No cases of HIV-HBV coinfection were evidenced in the studied population. HCV prevalence was low, with only one individual testing positive for anti-HCV antibodies, but with a negative confirmation test (undetectable HCV RNA).

**Table 3:** Prevalence of HIV, hepatitis B and hepatitis C *.

Condition—% (95% CI)	Total	Women	Men
Confirmed HIV infection	2.4 (1.3–4.2)	3.20 (1.6–6.1)	1.08 (0.04–4.1)
HBsAg positive	3.4 (2.1–5.5)	2.84 (1.4–5.6)	4.30 (2.1–8.4)
Anti-HBc (IgG + IgM) positive	36.5 (32.3–41.0)	34.8 (29.4–40.5)	39.3 (32.5–46.4)
Anti-HBc (IgM) positive	0.43 (0.0–1.7)	0.00 (0.0–1.6)	1.08 (0.04–4.1)
Confirmed hepatitis C virus infection	0.00 (0.0–0.9)	0.00 (0.0–1.6)	0.00 (0.00–4.1)

* Results were available for 466 subjects for HIV and 468 for all other samples.

Quality of life was surveyed using selected questions from the Chilean National Health Survey^19^. The results are detailed in Table 4. When asked how they felt about their life in general (work, family, wellbeing, health, love), on a scale from 1 to 7, the average response was 4.4 (34.9% responded less than regular, bad, or very bad). This was more frequent in subjects who arrived in Chile less than 1 year ago (< 1y) (52.7% vs 26.9%, p < 0.0001). Similarly, 29% felt a certain degree of sadness, or absolutely sad, down or depressed during the last month (35.7% in < 1y vs 21% in > 1y, p = 0.002), and 25.7% felt worried or anxious always or almost always (34% for < 1y vs 22% for > 1y, p = 0.008).

**Table 4:** Survey of quality of life and perception of health.

Question	Total	< 1 year in Chile	≥ 1 year in Chile
**"How do you feel in general (work. family. wellbeing. health. love)?"—n/N (%)**			
Very good	58/473 (12.3)	14/146 (9.6)	44/327 (13.5)
Good	181/473 (38.1)	41/146 (28.1)	140/327 (42.8)
More than regular	14/473 (3.0)	2/146 (1.4)	12/327 (3.7)
Regular	55/473 (11.6)	12/146 (8.2)	43/327 (13.1)
Less than regular	51/473 (10.8)	21/146 (14.4)	30/327 (9.2)
Bad	75/473 (15.9)	39/146 (26.8)	36/327 (11.0)
Very bad	39/473 (8.2)	17/146 (11.6)	22/327 (6.7)
**"In general. your health is:"—n/N (%)**			
Excellent	11/473 (2.3)	2/146 (1.4)	9/327 (2.8)
Very good	53/473 (11.2)	17/146 (11.6)	36/327 (11.0)
Good	214/473 (45.2)	57/146 (39.0)	157/327 (48.0)
Regular	104/473 (22.0)	26/146 (17.8)	78/327 (23.9)
Bad	91/473 (19.2)	44/146 (30.1)	47/327 (14.4)
**"During the last month. how frequently did you feel very energetic?"—n/N (%)**			
Always	84/473 (17.8)	17/146 (11.6)	67/327 (20.5)
Almost always	103/473 (21.8)	35/146 (24.0)	68/327 (20.8)
Sometimes	155/473 (32.8)	48/146 (32.9)	107/327 (32.7)
Rarely	115/473 (24.3)	42/146 (28.8)	73/327 (22.3)
Never	16/473 (3.4)	4/146 (2.7)	12/327 (3.7)
**"During the last month. how frequently did you feel sad or depressed?"—n/N (%)**			
Always	50/473 (10.6)	19/146 (13.0)	31/327 (9.5)
Almost always	73/473 (15.4)	33/146 (22.6)	40/327 (12.2)
Sometimes	149/473 (31.5)	42/146 (28.8)	107/327 (32.7)
Rarely	146/473 (30.9)	40/146 (27.3)	106/327 (32.4)
Never	55/473 (11.6)	12/146 (8.2)	43/327 (13.1)
**"During the last month. have you had discomfort or pain?"—n/N (%)**			
Always	66/473 (14.0)	10/146 (6.8)	56/327 (17.1)
Almost always	216/473 (45.7)	66/146 (45.2)	150/327 (45.9)
Sometimes	58/473 (12.3)	12/146 (8.2)	46/327 (14.1)
Rarely	122/473 (25.8)	55/146 (37.7)	67/327 (20.5)
Never	11/473 (2.3)	3/146 (2.1)	8/327 (2.4)
**"During the last month. how sad. down or depressed have you felt?"—n/N (%)**			
None	64/472 (13.6)	8/145 (5.5)	56/327 (17.1)
A little	218/472 (46.2)	65/145 (44.8)	153/327 (46.8)
Moderately	53/472 (11.2)	13/145 (9.0)	40/327 (12.2)
Pretty	127/472 (26.9)	53/145 (36.6)	74/327 (22.6)
Absolutely	10/472 (2.1)	6/145 (4.1)	4/327 (1.2)
**"During the last month. how often have you felt worried or anxious?"—n/N (%)**			
Never	75/471 (15.9)	12/144 (8.3)	63/327 (19.3)
Rarely	227/471 (48.2)	70/144 (48.7)	157/327 (48.1)
Sometimes	48/471 (10.1)	13/144 (9.0)	35/327 (10.7)
Almost always	102/471 (21.7)	43/144 (29.9)	59/327 (18.0)
Always	19/471 (4.0)	6/144 (4.2)	13/327 (4.0)
**"Have you ever been diagnosed with depression by a doctor or psychologist?"—n/N (%)**			
Yes	44/471 (9.3)	15/145 (10.3)	29/326 (8.9)
No	399/471 (84.7)	117/145 (80.7)	282/326 (86.5)
Don't know	28/471 (5.9)	13/145 (9.0)	15/326 (4.6)

## Discussion

International migration is generally thought as movement of people from developing nations to rich countries, so called South-North migration. Nevertheless, South-South migration (among developing countries), which accounts for as high as 33 to 45% of total migration^20^, is much less studied^21^. This kind of migration possesses different challenges, including inequality, poverty and xenophobia taking place in the context of host countries with generally weaker social and health public systems. The possible development implications of migration within the South has only recently being highlighted^22^, with most studies dealing with subjects such as labor and remittances, integration and social and public policies, but few studies focusing on health and well-being^23^.

Chile has become one of the preferred sites and the fastest-growing destination for international migration in South America, representing a case study for South-South migration. Chile typically attracts people from countries such as Perú, Bolivia, Colombia, and Venezuela, with comparable costumes, sharing the same language, and common Hispanic ethnicity. The recent arrival of a great influx of Haitian population to Chile, with a different language, African ancestry, distinct skin tone, and unique culture, represents a major challenge for integration. In this regard, a relevant aspect for successful integration is understanding the incoming population's unique characteristics, including their health status and needs. Unfortunately, very few studies, if any, have directly addressed the health status of the migrant populations entering and living in the country. Developing studies aimed towards understanding migrant communities is relevant to establish a comprehensive guideline to better welcome migrants by clearly identifying their specific needs. The present study provides an overview of the disease burden in Haitian migrants in Chile. With more than 2 million Haitians living abroad, objective information about Haitian migrants' health status is also relevant for other countries receiving this population.

The study shows that Haitian migrants in Chile are generally young (mean age 31 years old), healthy and slim. The prevalence of risk factors (such as obesity, hyperglycemia, smoking, or drinking) for developing chronic conditions was generally low, except for hypertension. Nutritional status assessed by BMI was normal in 45% of subjects, which is in sharp contrast to the Chilean population, with only 24.5% normal BMI^19^. Hypertension prevalence was strikingly high in the age group 25 to 44 years old (33% compared to 10.6% in the Chilean population^19^). This is consistent with reports showing that African descendants have a higher prevalence of hypertension, which develops at an earlier age, with higher average blood pressure and worse disease severity^24,25^.

The prevalence of three chronic viral infections was assessed in this study. Noteworthy, tuberculosis was not included in the survey as leaders of the Haitian community and NGOs associated with migration feared the stigma this information could bring to the migrant population. The prevalence of HIV infection was 2.4%, which is similar to what has been described in Haiti^26,27^, but strikingly higher than the HIV prevalence in Chile (0.36%)^28^. It should be noted that women had a higher prevalence (3.2%) compared with men (1.08%). This suggests that there are gender inequalities in this community that require further research, not included in this study^29^. Similarly, HIV prevalence was much higher in subjects with lower educational levels (Fig. 1b); 4% in subjects with primary education compared with 1.7 to 2.1% in participants with higher academic levels. This is interesting to note since there is not always an inverse relationship between educational levels and HIV prevalence^30^.

The estimated HBV infection was 3.4%, higher in men (4.3%) than in women (2.8%). By comparison, the prevalence of HBV infection in Chileans is 0.15%^31^. Total anti-HBc antibody, a marker of a previous infection, was 36.7%. Two subjects with an anti-HBc IgM antibody were detected among 16 HBsAg positive participants (12.5%), indicating acute infection. Both markers of infection (HBsAg and total anti-HBc) increased with age (Fig. 1c, e), suggesting that infection is an ongoing process during life, not just vertical transmission at birth or horizontal transmission at childhood. Alternatively, this may reflect the success of current vaccination programs in Haiti, as hepatitis B prevalence in children has recently reported being low (0.5%)^32^. These data underscore the need to address the issue of HBV transmission with public policy plans, such as voluntary screening programs, HBsAg detection during pregnancy, and vaccination for groups at risk. HBV vaccination is universal in Chile since 2006, but the calendar included the first dose at age 2 months. Fortunately, in 2019 the first dose was shifted to the time of birth, a move in the right direction, consistent with the current WHO recommendations^33^.

Migration may be a stressful and challenging decision for individuals and families. The impact of migration on mental health has been thoroughly described^34^. Our survey suggests significant anxiety and depression levels, which are more common in newer migrants (less than 1 year from arrival). Aside from the stress of the change, uncertainties of getting a job, and leaving the family, several other specific problems contribute to stress in this population: encountering a new, colder climate, facing a significant language barrier (67.1% had difficulty speaking Spanish) and racial discrimination. The impact of all these challenges in mental health, post-traumatic stress disorder, and mood disorders in this migrant population should be better characterized. When integrating the answers regarding the quality of life, employment, and the surprisingly high proportion of low BMI in men (9.1% compared to 1.3% in the Chilean population), results suggest that a proportion of these subjects included in the study are not receiving the minimum caloric intake for maintaining their weight.

The present study has some limitations. Sampling was not probabilistic, which may introduce selection bias. Several procedures (detailed in the methods section) were used to reduce potential bias. Selecting healthier participants could be favored if subjects within the community were aware of their health conditions (such as HIV infection) and were afraid of participating. However, this does not seem to be the case as the prevalence of HIV and HBV infection found in our sample was similar to what is described in Haiti. On the other hand, there is a risk of over-representing certain conditions if subjects with known diseases were more likely to participate. This risk was reduced by inviting subjects in communities instead of recruiting in health centers and avoiding giving participants the expectation of accessing treatment by being part of the study. Women were 60.4% of the sample, which is probably an over-representation of this population's true gender distribution.

Even with these limitations (a non-probabilistic sample), we believe that this study allows a better understanding of an understudied type of movement of people (South-South migration) and offers relevant and actionable health information regarding this particular population.

This survey allows us to conclude that Haitian migrants are generally healthy, with a lower prevalence of overweight and obesity, a low prevalence of diabetes, low frequency of smoking and alcohol drinking, and low cardiovascular risk. This better health profile could be explained by their younger age, or by a different, healthier diet and more physical activity, but diet was not directly assessed in this study. The high levels of sedentarism found suggest that physical activity was not the protective factor. The three main identified health problems are: (1) A higher prevalence of hypertension at a younger age, (2) high prevalence of HIV and HBV infection, which is consistent with the rates at their country of origin, and (3) markers suggesting a high frequency of anxiety and mental health disorders associated with migration. The generated information is useful not only to Chilean health authorities for planning public policies aiming at protecting the health and dignity of Haitian international migrants, but also for other countries receiving migrants from Haiti^35^. These policies should include aggressive detection and treatment of hypertension, offering screening programs for HIV and HBV infection, offering vaccination to seronegative subjects, and preparing a mental health program for Haitian immigrants.
